# Reply from authors: Preventing atrioesophageal fistula: A dish best served cold

**DOI:** 10.1016/j.xjtc.2022.04.025

**Published:** 2022-04-30

**Authors:** Barry C. Gibney, Ian C. Bostock

**Affiliations:** Division of Cardiothoracic Surgery, Department of Surgery, Medical University of South Carolina, Charleston, SC

Reply to the Editor:

**Figure:**
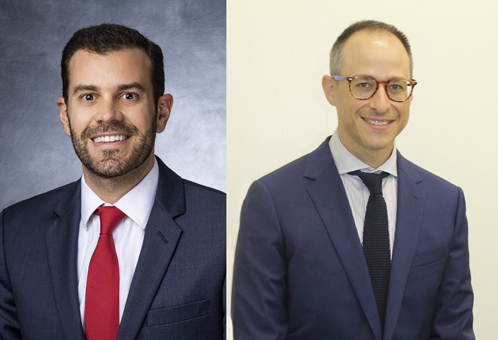

Dr Gibney is a consultant for Intuitive Surgical, Inc. Dr Bostock reported no conflicts of interest.

The *Journal* policy requires editors and reviewers to disclose conflicts of interest and to decline handling or reviewing manuscripts for which they may have a conflict of interest. The editors and reviewers of this article have no conflicts of interest.

Atrioesophageal (AE) fistula, while rare, represents a diagnostic and therapeutic challenge to surgeons and unfortunately carries a high mortality.^1^ Given the rarity of the disease, no true standard for repair exists, ranging from simple repair, to open approaches using cardiopulmonary bypass, and even esophageal exclusion. Certainly, prevention is the best medicine for this often-devastating complication.

Prevention has traditionally centered on the tenets of (1) real-time esophageal location—either with echocardiography or esophagography, (2) limiting energy delivery time with minimal contact force, (3) intraluminal temperature monitoring, and (4) prophylactic use of postablation proton pump inhibitors,^2^ even if efficacy hasn't been proven.^3^ In their letter to the editor, Joseph and colleagues^4^ inform the readership of a newer technique in the battle to prevent AE fistula—active cooling of the esophagus during thermal ablation. Supported by the IMPACT study, a single-center, double-blinded randomized trial enrolling 188 patients, the ensoETM device (Attune Medical) was used endoluminally to cool the esophagus to 4°C for at least 10 minutes, and at least until the ablation was completed. The primary end point analysis focused on the endoscopy findings, where the cooled patients showed a significant reduction in thermal injuries (17%), without changes in acute events, nor efficacy of ablation.^5^

While the IMPACT study was not designed to detect a reduction in AE fistula formation, the endoscopic findings certainly support the notion that endoscopic cooling would be beneficial. To that end, we agree with Joseph and colleagues that active cooling should be part of the tenets of prevention and absolutely advocated for by both electrophysiologists and cardiothoracic surgeons alike.
